# Outcomes of Combined Peritoneal and Local Treatment for Patients with Peritoneal and Limited Liver Metastases of Colorectal Origin: A Systematic Review and Meta-Analysis

**DOI:** 10.1245/s10434-021-10925-y

**Published:** 2021-10-22

**Authors:** Margot C. E. Polderdijk, Max Brouwer, Leonie Haverkamp, Kirsten A. Ziesemer, Mark Tenhagen, Djamila Boerma, Niels F. M. Kok, Kathelijn S. Versteeg, Dirkje W. Sommeijer, Pieter J. Tanis, Jurriaan B. Tuynman

**Affiliations:** 1 Department of Surgery, Amsterdam University Medical Centers, Amsterdam, The Netherlands; 2 Medical Library, Vrije Universiteit, Amsterdam, The Netherlands; 3 Department of Surgery, St. Antonius Hospital, Nieuwegein, The Netherlands; 4 Department of Surgery, Netherlands Cancer Hospital, Amsterdam, The Netherlands; 5 Department of Medical Oncology, Amsterdam University Medical Centers, Amsterdam, The Netherlands; 6 Department of Medical Oncology, Flevoziekenhuis, Almere, The Netherlands

## Abstract

**Background:**

Almost half of all colorectal cancer (CRC) patients will experience metastases at some point, and in the majority of cases, multiple organs will be involved. If the peritoneum is involved in addition to the liver, the current guideline-driven treatment options are limited. The reported overall survival ranges from 6 to 13 months for the current standard of care (systemic treatment). This study aimed to evaluate morbidity and clinical long-term outcomes from a combined local treatment of hepatic metastases with cytoreductive surgery (CRS) and hyperthermic intraperitoneal chemotherapy (HIPEC) used to treat peritoneal metastases.

**Methods:**

A systematic search was performed in PubMed, Embase.com, Web of Science, and Cochrane. Studies evaluating the clinicopathologic data of patients who had both peritoneal and hepatic metastases treated with CRS-HIPEC were included provided sufficient data on the primary outcomes (overall and disease-free survival) were presented. The quality of included studies was assessed using the Methodological Index for Non-Randomized Studies (MINORS).

**Results:**

Patients treated for peritoneal and liver metastases (PMLM group) had a pooled mean survival of 26.4 months (95% confidence interval [CI] 22.4–30.4 months), with a 3-year survival rate of 34% (95% CI 26.7–42.0%) and a 5-year survival rate of 25% (95% CI 17.3–33.8%). Surgical complications occurred more frequently for these patients than for those with peritoneal metastasis only (40% vs 22%; *p* = 0.0014), but the mortality and reoperation rates did not differ significantly.

**Conclusion:**

This systematic review showed that CRS and HIPEC combined with local treatment of limited liver metastasis for selected patients is feasible, although with increased morbidity and an association with a long-term survival rate of 25%, which is unlikely to be achievable with systemic treatment only.

**Supplementary Information:**

The online version contains supplementary material available at 10.1245/s10434-021-10925-y.

Colorectal cancer (CRC) is the third most common cancer in the world and the second cause of cancer-related death, with almost 2 million new cases worldwide every year.^1^ Metastases are present at diagnosis in 20 to 25% of cases, and for another 20 to 25% of patients, metastatic disease develops after initial surgical treatment.^2,3^ For the majority of these patients, multiple organs are involved.^4^ The liver is most commonly affected, followed by the peritoneum and lungs.^5^ When distant metastases are present, the quantity and location significantly affect prognosis, as well as the suitability for local treatment. For patients with liver lesions amenable to local ablative and/or surgical treatment, 5-year overall survival (OS) rates of more than 60% have been reported.^6–10^

Patients with peritoneal metastases (PM) from colorectal cancer have a considerably worse prognosis. Without treatment, these patients often do not survive longer than 12 months.^11^ Systemic therapy for peritoneal metastasis has limited effects, increasing median survival from 12 to 16 months.^12^ The introduction of cytoreductive surgery (CRS) and hyperthermic intraperitoneal chemotherapy (HIPEC) has resulted in favorable survival rates for selected patients who have limited disease confined to the abdominal cavity, with a median OS of 20 to 63 months^13^ and 5-year OS rates ranging from 23 to 52%.^14^ The survival benefit of this procedure is dependent on the severity of disease, represented by the Peritoneal Cancer Index (PCI), the completeness of cytoreduction (CC), and the histopathology of the tumor.^13^ These factors form the basis for the procedure’s current eligibility criteria specifying that the extent of peritoneal disease must not exceed a certain PCI (commonly used in practice: PCI < 16–20), that adequate cytoreduction must be possible, and that signet ring cell histopathology must be a relative contraindication.

Currently, synchronous hematogenous liver, lung, or other distant metastases generally are considered a contraindication for CRS-HIPEC.^15^ For patients who do not meet the selection criteria for CRS-HIPEC, disease generally is considered non-curable. Therefore, palliative systemic therapy is their sole treatment option.

Currently, centers demonstrate large heterogeneity in whether combined CRS-HIPEC and local ablation of liver metastases is offered. If it is offered, CRS-HIPEC is performed only for selected cases (e.g., for young, fit patients with a low PCI and only one or two liver lesions without signet ring cell histology). Cohort studies have shown beneficial effects of HIPEC combined with surgical/radiologic ablation of liver metastasis for these highly selected patients. A meta-analysis by Hallam et al.^13^ has shown that surgically treated hepatic metastasis is not predictive for overall survival after a CRS-HIPEC procedure. In 2007, Esquivel et al.^16^ released a consensus statement compiled in collaboration with field experts from all around the world asserting that a patient may be considered for CRS-HIPEC when no more than three resectable, small hepatic lesions are present. However, due to lack of robust data, current guidelines do not offer clinical directives toward local treatment for patients with hepatic oligometastases and peritoneal metastases. Consequently, palliation with systemic therapy still is considered the only meaningful standard therapy.

This systematic review and meta-analysis of the literature aimed to determine the feasibility and clinical long-term outcome of CRS-HIPEC combined with local treatment for patients with both peritoneal and limited hepatic metastases.

## Methods

The performance and reporting of this systematic review adhere to the Preferred Reporting Items for Systematic Reviews and Meta-Analyses (PRISMA) statement (www.prisma-statement.org).^17^ The review protocol was registered at PROSPERO (www.crd.york.ac.uk/PROSPERO), where it can be found under registration number CRD42021219836.

### Search Strategy

After scoping searches, four bibliographic databases (PubMed, Embase.com, Clarivate Analytics/Web of Science Core Collection, Wiley/Cochrane Library) were searched systematically for relevant literature from inception of the review to 9 November 2020. The searches were devised in collaboration with a medical information specialist (K.A.Z.). Search terms including synonyms, closely related words, and keywords such as “metastatic colorectal cancer” and “HIPEC” were used as index terms or free-text words. The searches contained no search filter, date, or language restrictions that would limit results to a specific study design, date, or language. Duplicate articles were excluded using Endnote (X9.3.3, AED method and Bramer method).^18,19^ The full search strategy used for each database is outlined in the supplementary material (Tables 1, 2, 3, and 4). In addition to the database searches, reference lists of the included full-text articles were screened to identify additional relevant articles.

**Table 1:** Characteristics of included studies

No.	First author (year)	Period	Study type	*n*	Groups	Median PCI	Synchronous : metachronous	Preoperative chemotherapy (%)	Postoperative chemotherapy (%)	Complete cytoreduction (CC0/R1) (%)	HIPEC agent	MINORS
1	Adileh^23^	2007–2019	Prospective cohort	140	PMLM: 60 PM: 80	11 8	NR	97 100	NR	100 100	MMC	15/24
2	Alzahrani^24^	2003–2014	Prospective cohort	78	PMLM: 36 PM: 42	7 12	NR	92 41	92 81	97 93	MMC / OX	15/24
3	Baratti^25^	2004–2016	Prospective cohort	148	PM+EPD^i^: 27 PM: 121	8.5 10	1 : 1	81 65	NR	85 78	MMC ± cisplatin	17/24
4	Berger^26^	2007–2014	Retrospective cohort	269	PMLM: 103 PM: 166	18.5 7	NR	58 57	NR	83.5^c^ 81.8	MMC	15/24
5	Delhorme^27^	2007–2011	Prospective cohort	27	PMLM: 9 PM: 18	19 9	2 : 1	100 100	33 28	100 100	MMC or OX	17/24
6	Downs-Canner^28^	2005–2013	Prospective cohort	205	PMLM: 32 PM: 173	13.7^a^ 11.2^a^	NR	97 NR	69 63	84 84	MMC	17/24
7	Duraj^29^	1994–2010	Case-control	33	PMLM: 11 PM: 22	13^b^	2.3 : 1	90 59	36 32	91 91	OX ± IRI	18/24
8	Horvath^30^	2006–2016	Prospective cohort	37	PMLM: 37	14	NR	78	NR	NR	Cisplatin / MMC / OX	9/16
9	Jeon^31^	2014–2018	Retrospective cohort	22	PMLM: 22	13	1 : 1.2	91	100	100	MMC	9/16
10	Kianmanesh^32^	1992–2005	Retrospective cohort	43	PMLM: 16 PM: 27	NR	1 : 1.4	70^b^	75^b^	70^b^^,c^	MMC + cisplatin	6/16
11	Lee^33^	2000–2017	Retrospective cohort	658	PMLM: 83 PM: 575	12.8^a^ 12.8^a^	Synchronous	59 37	NR	NR	NR	14/24
12	Lo Dico^34^	1993–2017	Prospective cohort	534	1-step: 437 Liver-first: 66 HIPEC-first: 31	10.1^a^ 9.1^a^ 7.6^a^	1 : 1.5	78 65 66	57 36 48	91 70 87	MMC + cisplatin	10/16
13	Lorimier^35^	1999–2011	Prospective cohort	58	PMLM: 22 PM: 36	15 10.5	NR	86 86	86 86	86 69	MMC + cisplatin / OX	17/24
14	Maggiori^36^	1993–2009	Case-control	98	PMLM: 37 PM: 61	11 9	NR	NR	81 79	100 100	MMC + cisplatin	18/24
15	Morales Soriano^37^	2010–2015	Prospective cohort	61	PMLM: 16 PM: 45	10.6^a^ 9.9	NR	81 73	NR	94 93	OX or MMC	17/24
16	Mouw^38^	2005–2016	Retrospective cohort	43	PMLM: 20 PM: 23	NR	Synchronous	NR	NR	100 82	NR	17/24
17	Navez^39^	2007–2015	Retrospective cohort	77	PMLM: 25 PM: 52	10 6	1.6 : 1	84 73	52 88	100 100	OX or MMC	16/24
18	Pinto^40^	2007–2016	Retrospective cohort	109	PMLM: 33 PM: 76	9 6	1.75 : 1	100 88	56 11	67 76	OX	15/24
19	Randle^41^	1991–2013	Prospective cohort	233	PMLM: 32 PM: 201	NR	NR	100 100	NR	53 53	NR	18/24
20	Saxena^42^	1996–2015	Prospective cohort	264	PMLM: 66 PM: 198	6^b^	NR	NR	NR	NR	NR	17/24

HIPEC, hyperthermic intraperitoneal chemotherapy; MINORS, Methodological Index for Non-Randomized Studies; PMLM, peritoneal and liver metastases; NR, not reported; MMC, mitomycin C; OX, oxaliplatin; PM, peritoneal metastases; EPD, extraperitoneal disease; IRI, irinotecan

^a^Mean instead of median

^b^Overall (mean)

^c^CC0 & CC1

**Table 2:** Liver treatment details

First author (year)	No. of liver lesions	LM treatment	Preoperative chemotherapy (%)
Adileh^23^	NR	Resection	97
Alzahrani^24^	< 3: 25 ≥ 3: 11	Resection	92
Baratti^25^	Mean 2.38	Resection and/or RFA	81
Berger^26^	NR	Resection	58
Delhorme^27^	Median 1	Resection and/or RFA	100
Downs-Canner^28^	1: 16 pts 2: 7 pts 3 or more: 7	Resection and/or RFA	97
Duraj^29^	Median 1	Resection	90
Horvath^30^	1–2: 24 pts > 2: 4 pts	Resection	78
Jeon^31^	Median: 3	Resection and/or RFA	91
Kianmanesh^32^	NR	Resection	70^a^
Lee^33^	NR	Resection	59
Lo Dico^34^	Median: 1	Resection	76
Lorimier^35^	Mean 1.9	Resection and/or RFA	86
Maggiori^36^	Median: 2	Resection and/or RFA	NR
Morales Soriano^37^	Mean 1.2	Resection and/or RFA	81
Mouw^38^	NR	resection	NR
Navez^39^	≤ 3	Resection and/or RFA	84
Pinto^40^	NR	Resection and/or RFA	100
Randle^41^	NR	NR	100
Saxena^42^	1: 34 pts 2–3: 30 pts 4 or more: 6	NR	NR

LM, local metastasis; NR, not reported; RFA, radiofrequency ablation; pts, patients

^a^Overall

**Table 3:** Long-term oncologic outcomes for the PMLM group

Outcome	Pooled mean (95% CI)	No. of studies	No. of patients
DFS (months)	10.8 (8.0–13.6)	10	281
3-Year DFS (%)	14.4 (8.3–23.8)	10	276
Recurrence rate (%)			
Overall	71.8 (64.7–77.9)	11	809
Peritoneum	32.4 (26.4–39.2)	9	763
Liver	33.2 (26.8–40.4)	9	763
Lung	23.1 (18.1–29.0)	6	683
Other	15.9 (9.0–26.6)	8	752
3-Year survival (%)	33.9 (26.7–42.0)	13	406
5-Year survival (%)	24.6 (17.3–33.8)	12	384
OS (months)	26.4 (22.4–30.4)	14	399

PMLM, peritoneal and liver metastases; CI, confidence interval; DFS, disease-free survival; OS, overall survival

**Table 4:** Disease-free survival at the 5-year follow-up evaluation

Study	*n*/total (%)
Delhorme^27^	1/9 (11.1)
Downs-Canner^28^	2/32 (6.3)
Lorimier^35^	0/22 (0)
Maggiori^36^	1/37 (2.7)
Pinto^40^	8/33 (24.2)
Pooled mean:% (95% CI)	6.3 (2.0–18.6)

CI, confidence interval

### Screening Process

One reviewer (M.P.) screened all potentially relevant titles and abstracts for eligibility, selecting those that described patients treated with CRS-HIPEC who had metastases at sites other than the peritoneum. Each full-text article then was independently screened by at least two members of the review team (M.P., M.B., L.H.). Studies were included if they met the criteria, which specified (1) types of studies (randomized control studies [RCTs], cohort studies, case-control studies, and cross-sectional studies; (2) types of participants (patients with primary colorectal cancer metastasized to the peritoneum and one hematogenous site); and (3) types of interventions (studies that involved multimodal treatment with both CRS-HIPEC and a local [ablative] treatment). Any local treatment performed with the intention to remove or eradicate all tumor cells was accepted. The review excluded letters to the editor, animal studies, *in vitro*/*ex vivo* studies, case reports, (systematic) reviews, and meta-analyses. Studies reporting on fewer than 10 patients also were excluded from the analysis. The location and study period of all the included articles were compared to identify duplicate data, and if (partially) overlapping data were found, only the study describing the largest group of patients was included.

### Data Extraction and Quality Assessment

Data were collected from the included articles using a data extraction form. The completed forms were used to pool the data and to perform statistical analyses. Based on the collected data, the results were organized into two groups: a group of patients with peritoneal metastasis (PM) only and a group of patients with PM and simultaneous liver metastasis (PMLM).

The main outcomes of the analysis were overall survival (OS) and disease-free survival (DFS), calculated from the date of CRS-HIPEC. The secondary end points were perioperative outcomes, including morbidity and mortality. Major morbidity was defined as the presence of a complication classified as Clavien-Dindo grade 3 or higher.

For a proper comparison of studies, data on follow-up length, CC, PCI, and pre- and postoperative chemotherapy also were recorded. Risk of bias in individual studies was assessed using the Methodological Index for Non-Randomized Studies (MINORS).^20^

### Statistical Analysis

Meta-analysis was performed for both perioperative and survival outcomes in the PMLM group. To determine the feasibility of the combined treatment, a meta-analysis of perioperative outcomes for the PM group was performed using formal statistical comparison with the PMLM group. Only studies that provided appropriate data were included in the meta-analysis for perioperative outcomes.

Major morbidity and mortality risk ratios (RRs) were calculated using the number of events and the numbers of patients at risk, which also was how proportional survival outcomes such as 3- and 5-year overall survival data were pooled. For the survival outcomes, measured in months, means, and standard deviations, were calculated using the method described by Wan et al.,^21^ after which these means were pooled. This was performed only for the PMLM group due to the objective of this study. A random-effects model was applied in all analyses due to large heterogeneity. All analyses were performed using the meta package in R.^22^

## Results

### Study Selection

**Fig. 1:**
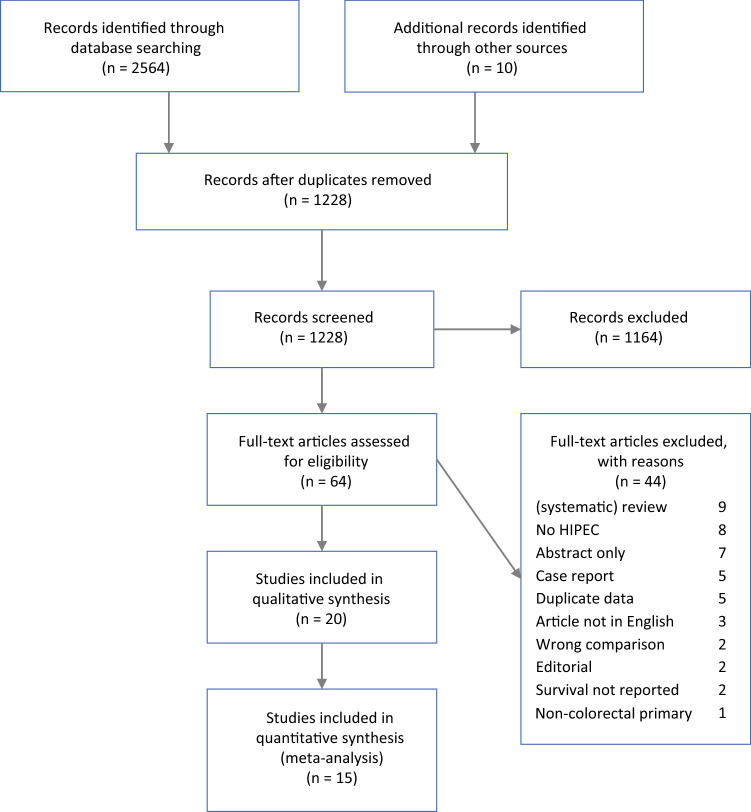
Search and selection process (PRISMA flowchart)

The literature search generated 2621 references: 463 in PubMed, 1403 in Embase.com, 698 in Clarivate Analytics/Web of Science Core Collection, and 57 in Wiley/Cochrane library. After removal of reference duplicates selected from more than one database, 1218 references remained, all of which were included in the title/abstract screening. Of these articles, 54 were included for full-text assessment, together with an additional 10 articles identified from their reference lists. This resulted in 20 articles included for the qualitative synthesis, 16 of which presented sufficient data to be included in the quantitative synthesis. The search and selection process is illustrated in Fig. 1.

### Study Characteristics

In total, 3137 patients participated in 2 case-control studies, 11 prospective studies, and 7 retrospective cohort studies.^23–42^ No randomized studies were identified. Although the search and selection process was designed to include various oligometastatic sites, the studies that met the criteria for inclusion all presented liver metastases. With the exception of the studies by Baratti et al.^25^ and Lo Dico et al.,^34^ the studies in this review exclusively presented patients treated in a one-step procedure, with CRS-HIPEC and liver resection/ablation performed during one surgical procedure.

Relatively high variability in PCI, CC, and perioperative systemic therapy protocols was found among the studies (Table 1). In most of the studies, the PMLM patients had a higher PCI than the PM-only patients. In the vast majority of the studies, the PCI was below 20, which corresponds with clinical guidelines. The CC rates varied between 53 and 100%, with a mean of 88% in the PMLM group and 80% in the PM-only group. The majority of the participants received pre- and/or postoperative chemotherapy, with a higher proportion in the preoperative setting for the PMLM group ((78% vs 62%) and similar percentages in the PM-only group (60% vs 61%). The most frequently used chemotherapeutic agent for HIPEC was mitomycin C, either with or without cisplatin, followed by oxaliplatin.

### Survival Outcomes

Only a small proportion of the trials (*n* = 13) reported the extent of liver disease. Liver disease was limited in the majority of patients, as described by either a low number of lesions or a limited resection. The methods used to treat the hematogenous metastases included resection and radiofrequency ablation (RFA). Details of the liver treatment are presented in Table 2.

Table 3 shows data on the long-term oncologic outcomes in the PMLM group. Overall, 72% of the patients experienced recurrence. These recurrences most frequently occurred in the peritoneum (32%) or liver (33%), followed by pulmonary dissemination (23%), and 16% of the recurrences developed at another site. The pooled mean time to detection of these recurrences (DFS) was 10.8 months (95% confidence interval [CI] 8.0–13.6 months).

After 3 years, a pooled proportion of 14% of the patients (95% CI 8.3–23.8%) were disease-free (13 studies involving 406 patients). A small percentage of the patients (6%) still showed no evidence of disease after 5 years, as shown in Table 4. The pooled mean overall survival was 26.4 months (95% CI 22.4–30.4 months), and the pooled 3- and 5-year overall survival rates were 34% and 25%, respectively. Finally, Table 5 shows an overview of the PCI, CC score, and median OS for all the studies.

**Table 5:** Overview of PCI, CC score, and OS for both groups

Study	Group	Median PCI	Complete cytoreduction (CC0/R1) (%)	Median OS
Alzahrani^24^	PMLM: 36 PM: 42	7 12	97 93	24.4 45.5
Baratti^25^	PM+EPD: 27 PM: 121	8.5 10	85 78	19.0 60.1
Berger^26^	PMLM: 103 PM: 166	18.5 7	83.5^a^ 81.8	45.1^b^ 73.5^b^
Delhorme^27^	PMLM: 9 PM: 18	19 9	100 100	27.6 39.1
Downs-Canner^28^	PMLM: 32 PM: 173	13.7^c^ 11.2^c^	84 84	13 20.5
Duraj^20^	PMLM: 11 PM: 22	13^d^	91 91	15 34
Horvath^30^	PMLM: 37	14	NR	22
Jeon (2019)	PMLM: 22	13	100	16.7
Kianmanesh^32^	PMLM: 16 PM: 27	NR	70^a^^,d^	36.0 35.3
Lo Dico^34^	1-step: 437 Liver-first: 66 HIPEC-first: 31	10.1^c^ 9.1^c^ 7.6^c^	91 70 87	44.8^b^ 63.7^b^ 52.6^b^
Lorimier^35^	PMLM: 22 PM: 36	15 10.5	86 69	36.1^b^ 25.2^b^
Maggiori^36^	PMLM: 37 PM: 61	11 9	100 100	32 49
Morales Soriano^37^	PMLM: 16 PM: 45	10.6^c^ 9.9	94 93	36 33
Navez^39^	PMLM: 25 PM: 52	10 6	100 100	27.5 59.2
Pinto^40^	PMLM: 33 PM: 76	NR	67 76	31 65
Randle^41^	PMLM: 32 PM: 201	NR	53 53	21.2 33.6
Saxena^42^	PMLM: 66 PM: 198	6^d^	NR	32.3 30.5

PCI, peritoneal cancer index; CC, completeness of cytoreduction; OS, overall survival; PMLM, peritoneal and liver metastases; PM, peritoneal metastases; EPD, extraperitoneal disease NR, not reported; HIPEC, hyperthermic intraperitoneal chemotherapy

^a^CC0 & CC1

^b^Calculated from date of diagnosis

^c^Mean instead of median

^d^Overall (mean)

### Perioperative Outcomes

As shown in Table 6, major morbidity due to surgery was reported in 11 studies, with rates ranging from 12 to 75% in the PMLM group versus 9% to 40% in the PM-only group, and averages of 40% and 22%, respectively (RR, 1.78; *p* = 0.0014). Pooled mortality tended to be higher in the PMLM group (5% vs 2%; *p* = 0.0956), but the difference was not significant, similar to the reoperation rate (21% vs 16%; *p* = 0.2744). The mean hospital and intensive care unit (ICU stays could not be reliably calculated due to the confounding effect of outliers in the individual study data. The median hospital stay ranged from 13 to 28 days in the PMLM group and from 9 to 25 days in the PM group. The ICU stay was 1 to 12 days in the PMLM group and 1 to 16 days in the PM group.

**Table 6:** Perioperative outcomes^a^

Outcome	PMLM		PM		RR	*p* Value
	% (95% CI)	*n*	% (95% CI)	*n*		
Major morbidity	39.9 (29.2–51.7)	291	22.0 (16.7–28.4)	688	1.78	0.0014
Mortality	5.3 (2.9–9.6)	198	2.0 (0.8–4.7)	532	2.7	0.0956
Reoperation	21.2 (16.0–27.6)	189	16.0 (9.0–27.0)	921	1.38	0.2744

PMLM, peritoneal and liver metastases; PM, peritoneal metastases; RR, risk ratio

^a^Data are displayed as pooled mean (95% confidence interval)

## Discussion and Conclusion

This systematic review and meta-analysis show that combined local treatment of peritoneal and hepatic metastases for selected patients is feasible, resulting in a pooled mean overall survival period of 26.4 months and a pooled 5-year overall survival rate of 25%. These survival outcomes suggest an improvement compared with literature reporting on similar patients who received only systemic therapy,^11,12,43,44^ although a formal comparison could not be made because the available literature lacked studies comparing combined local treatment and systemic therapy. These data provide a lead to justify combined CRS-HIPEC and local ablative treatment for patients with both limited liver and peritoneal metastasis as an alternative for systemic treatment only and form a basis for prospective studies that further elucidate the role of local treatment of combined metastatic sites.

The specific group of patients who have both peritoneal and limited hepatic lesions treated with current standard systemic therapy is not well described in literature, and no comparative studies with surgical treatment are available at all. Several non-comparative studies describe patients treated with systemic therapy for peritoneal dissemination and any amount of systemic disease, without specification of location or extent. Pooled individual data of these patients from the Analysis and Research in Cancers of the Digestive System (ARCAD) database, which contains data from multiple randomized chemotherapy trials, show a median survival period of 12.6 months (95% CI 12.0–13.1 months).^12^

Another large population-based study by Simkens et al.^11^ that included almost 2500 patients with peritoneal and systemic metastases reported a median survival range of 6.8 to 13.3 months for patients treated with systemic therapy, depending on tumor histology. Furthermore, Pelz et al.^43^ and Razenberg et al.^44^ presented results with a median survival of 8.0 to 9.9 months for a mixed treatment population.

It should be noted that the average disease burden (regarding location and volume) in these groups likely was higher than in the studies included in this review of patients who received surgical treatments. In addition, younger patients in good physical condition tend to be less likely to receive systemic therapy only. These two factors resulted in an underestimation of survival in the systemic treatment group. Nevertheless, data from the current review showed a survival period of 26.4 months for the patients treated with CRS-HIPEC and local treatment (liver resection/radiofrequency ablation [RFA] or other ablative techniques), which at least warrants further investigation and indicates that a direct comparison is needed to determine whether an actual survival benefit exists. In addition, several studies included in this review showed long-term DFS for a small number of patients, which is unlikely to occur with systemic therapy alone.

The survival benefit is countered by the treatment-associated morbidity. Systemic therapy usually is a long-term process, during which complications might develop and worsen over time, whereas surgical complications typically are more short-term but more intense and life-threatening. As such, it is difficult to compare the burden of systemic treatment with that of surgery because the inherent risks differ in nature. Instead, a fairer comparison for determining the hazards of combined treatment is between CRS-HIPEC only and CRS-HIPEC with additional liver treatment (RFA and/or resection).

This study showed that the addition of liver resection and ablation is associated with increased morbidity (RR, 1.78; *p* = 0.0014). The differences in reoperation and mortality rates, however, were not found to be significant. For patients with metastasis limited to the peritoneum, the morbidity rate for CRS-HIPEC is high but accepted by clinicians and patients because of the long-term survival benefits. Based on the results of the current study, the same could be argued for the combined treatment. Furthermore, although more complications occurred, the range of median hospital stays in the PMLM group differed only a couple of days from that in the PM-only group.

This systematic review provides an extensive overview of the current literature. However, the current literature is hampered by the quality of the data. The studies included in this review used different criteria for inclusion and exclusion of patients, and potential selection bias could not be excluded. Although not always formally asserted, physical fitness of the patient was an important factor in determining eligibility for combined treatment.

Other inclusion criteria were quite heterogeneous, which was a limitation of the current review. An example of such a criterion was the extent of liver disease. As shown in Table 2, the hepatic disease burden was low on the average, but no clear cutoff value was universally applied. This should also be a point of focus in future investigations. Especially noteworthy was the variation in the use of preoperative chemotherapy. Eight of the included studies^24,28,30,34–37,40^ included only patients who did not show disease progression during or after preoperative systemic therapy. In other studies, this was not required, resulting in a mixed population of responders, non-responders, and patients who did not receive any preoperative chemotherapy.

In addition, the use of postoperative chemotherapy varied considerably between studies. These parameters likely influenced survival, thus resulting in heterogeneous outcomes among the various cohorts. The same could be argued about CC, which has been identified as a prognostic factor for survival.^13^

Some retrospective studies limited their participants to patients with complete cytoreduction (CC-0), but this was not possible for the prospective studies. A low percentage of CC-0 within a cohort could negatively influence survival data and affect the reported outcome.

The most important weakness of the included cohort studies was the improper group comparison. Preferably, palliative systemic treatment would be compared with extensive ablative treatment for these patients, but unfortunately, such studies have not been published to date, and thus, only an indirect comparison based on the literature was possible.

In conclusion, the current study showed that it is feasible to perform CRS-HIPEC and liver resection and/or ablation for patients with both peritoneal and limited hepatic metastasis because it seems to provide favorable long-term clinical outcomes despite current international guidelines suggesting that CRS-HIPEC not be performed for these patients. However, this combined treatment needs to be more closely investigated in future trials. Participants should be selected carefully based on their physical condition and disease burden, for which specific criteria have yet to be determined. This should be considered carefully in the design of studies confirming these data for safe implementation of the combined CRS-HIPEC and liver metastasis treatment for selected patients in clinical practice.

## Supplementary Information

Below is the link to the electronic supplementary material.

Supplementary file1 (DOCX 28 KB)
