# Supplementary material for: Outcomes of Combined Peritoneal and Local Treatment for Patients with Peritoneal and Limited Liver Metastases of Colorectal Origin: A Systematic Review and Meta-Analysis
Source: Ann Surg Oncol. 2021 Oct 22;29(3):1952–62. doi: 10.1245/s10434-021-10925-y (PMC8810452; doi:10.1245/s10434-021-10925-y)
Supplement: Supplementary file 1 — Supplementary file1 (DOCX 28 KB) [file 10434_2021_10925_MOESM1_ESM.docx]

**Supplementary material**

**Table 1: PubMed 463 results (9 November, 2020)**

| **Search** | **Query** | **Results** |
| --- | --- | --- |
| 3 | #1 AND #2 | 463 |
| 2 | "HIPEC*"[tiab] OR "IPC"[tiab] OR "Hyperthermic Intraperitoneal Chemotherap*"[tiab] OR "hyperthermic intra-peritoneal chemotherap*"[tiab] OR "peritoneal chemotherap*"[tiab] | 6,873 |
| 1 | ("Neoplasm Metastasis"[Mesh] OR "metasta*"[tiab]) AND ("Colorectal Neoplasms"[Mesh:NoExp] OR "Colonic Neoplasms"[Mesh] OR "Rectal Neoplasms"[Mesh:NoExp] OR "colorectal cancer*"[tiab] OR "colorectal carcinoma*"[tiab] OR "colorectal tumor*"[tiab] OR "colorectal tumour*"[tiab] OR "colorectal neoplas*"[tiab] OR "colonic cancer*"[tiab] OR "colonic carcinoma*"[tiab] OR "colonic tumor*"[tiab] OR "colonic tumour*"[tiab] OR "colonic neoplas*"[tiab] OR "colon cancer*"[tiab] OR "colon carcinoma*"[tiab] OR "colon tumor*"[tiab] OR "colon tumour*"[tiab] OR "colon neoplas*"[tiab] OR "rectum cancer*"[tiab] OR "rectum carcinoma*"[tiab] OR "rectum tumor*"[tiab] OR "rectum tumour*"[tiab] OR "rectum neoplas*"[tiab] OR "rectal cancer*"[tiab] OR "rectal carcinoma*"[tiab] OR "rectal tumor*"[tiab] OR "rectal tumour*"[tiab] OR "rectal neoplas*"[tiab]) OR (("advanced colorectal cancer*"[tiab] OR "advanced colon cancer*"[tiab] OR "advanced rectum cancer*"[tiab] OR "advanced colorectal carcinoma*"[tiab] OR "advanced colon carcinoma*"[tiab] OR "advanced rectum carcinoma*"[tiab] OR "advanced rectal cancer*"[tiab] OR "advanced rectal carcinoma*"[tiab]) NOT ("locally advanced colorectal cancer*"[tiab] OR "locally advanced colon cancer*"[tiab] OR "locally advanced rectum cancer*"[tiab] OR "locally advanced colorectal carcinoma*"[tiab] OR "locally advanced colon carcinoma*"[tiab] OR "locally advanced rectum carcinoma*"[tiab] OR "locally advanced rectal cancer*"[tiab] OR "locally advanced rectal carcinoma*"[tiab])) | 62,041 |

**Table 2: Embase.com 1403 results, 959 excluding conference abstracts (9 November, 2020)**

| **No.** | **Query** | **Results** |
| --- | --- | --- |
| #4 | #3 NOT 'conference abstract'/it | 959 |
| #3 | #1 AND #2 | 1403 |
| #2 | 'hyperthermic intraperitoneal chemotherapy'/exp OR 'hipec*':ti,ab,kw OR 'ipc':ti,ab,kw OR 'hyperthermic intraperitoneal chemotherap*':ti,ab,kw OR 'hyperthermic intra-peritoneal chemotherapy*':ti,ab,kw OR 'peritoneal chemotherapy*':ti,ab,kw | 11120 |
| #1 | ('metastatic colorectal cancer'/exp OR 'metastatic colorectal cancer' OR 'metastasis'/exp OR 'metastasis' OR 'metasta*':ti,ab,kw) AND ('colon tumor'/exp OR 'colon tumor' OR 'rectum tumor'/exp OR 'rectum tumor' OR (((('colorectal' OR 'colonic' OR 'colon' OR 'rectum' OR 'rectal') NEAR/3 ('cancer*' OR 'carcinoma*' OR 'tumor*' OR 'tumour*' OR 'neoplas*')):ab,ti,kw) NOT ('locally advanced colorectal cancer*':ab,ti,kw OR 'locally advanced colon cancer*':ab,ti,kw OR 'locally advanced rectum cancer*':ab,ti,kw OR 'locally advanced colorectal carcinoma*':ab,ti,kw OR 'locally advanced colon carcinoma*':ab,ti,kw OR 'locally advanced rectum carcinoma*':ab,ti,kw OR 'locally advanced rectal cancer*':ab,ti,kw OR 'locally advanced rectal carcinoma*':ab,ti,kw))) | 110112 |

**Table 3: Clarivate Analytics/Web of Science Core Collection 698 results (9 November, 2020)**

| **Query** | **Result** | **Details** |
| --- | --- | --- |
| \| # 8 \| \| --- \| \| | [698](http://apps.webofknowledge.com.vu-nl.idm.oclc.org/summary.do?product=WOS&doc=1&qid=17&SID=F3hBnvbSnv7HrsVeRHI&search_mode=AdvancedSearch&update_back2search_link_param=yes) | #6 AND #7 |
|  |  | *Indexes=SCI-EXPANDED, SSCI, A&HCI, ESCI Timespan=All years* |
| \| # 7 \| \| --- \| \| | [9,524](http://apps.webofknowledge.com.vu-nl.idm.oclc.org/summary.do?product=WOS&doc=1&qid=16&SID=F3hBnvbSnv7HrsVeRHI&search_mode=AdvancedSearch&update_back2search_link_param=yes) | TS=(“HIPEC*” OR “IPC” OR “Hyperthermic intraperitoneal Chemotherap*” OR “Hyperthermic intra-peritoneal chemotherapy*” OR “peritoneal chemotherapy*”) |
|  |  | *Indexes=SCI-EXPANDED, SSCI, A&HCI, ESCI Timespan=All years* |
| \| # 6 \| \| --- \| \| | [70,785](http://apps.webofknowledge.com.vu-nl.idm.oclc.org/summary.do?product=WOS&doc=1&qid=15&SID=F3hBnvbSnv7HrsVeRHI&search_mode=AdvancedSearch&update_back2search_link_param=yes) | TS=(metasta*) AND TS=((colorectal OR colonic OR colon OR rectum OR rectal) NEAR/3 (cancer* OR carcinoma* OR tumor* OR tumour* OR neoplas*) ) NOT TS=(“locally advanced colorectal cancer*“ OR “locally advanced colon cancer*“ OR “locally advanced rectum cancer*“ OR “locally advanced colorectal carcinoma*“ OR “locally advanced colon carcinoma*“ OR “locally advanced rectum carcinoma*“ OR “locally advanced rectal cancer*“ OR “locally advanced rectal carcinoma*“) |
|  |  | *Indexes=SCI-EXPANDED, SSCI, A&HCI, ESCI Timespan=All years* |
| \| # 5 \| \| --- \| \| | [70,785](http://apps.webofknowledge.com.vu-nl.idm.oclc.org/summary.do?product=WOS&doc=1&qid=14&SID=F3hBnvbSnv7HrsVeRHI&search_mode=AdvancedSearch&update_back2search_link_param=yes) | #3 NOT #4 |
|  |  | *Indexes=SCI-EXPANDED, SSCI, A&HCI, ESCI Timespan=All years* |
| \| # 4 \| \| --- \| \| | [3,915](http://apps.webofknowledge.com.vu-nl.idm.oclc.org/summary.do?product=WOS&doc=1&qid=13&SID=F3hBnvbSnv7HrsVeRHI&search_mode=AdvancedSearch&update_back2search_link_param=yes) | TS=(“locally advanced colorectal cancer*“ OR “locally advanced colon cancer*“ OR “locally advanced rectum cancer*“ OR “locally advanced colorectal carcinoma*“ OR “locally advanced colon carcinoma*“ OR “locally advanced rectum carcinoma*“ OR “locally advanced rectal cancer*“ OR “locally advanced rectal carcinoma*“) |
|  |  | *Indexes=SCI-EXPANDED, SSCI, A&HCI, ESCI Timespan=All years* |
| \| # 3 \| \| --- \| \| | [71,578](http://apps.webofknowledge.com.vu-nl.idm.oclc.org/summary.do?product=WOS&doc=1&qid=8&SID=F3hBnvbSnv7HrsVeRHI&search_mode=AdvancedSearch&update_back2search_link_param=yes) | #1 AND #2 |
|  |  | *Indexes=SCI-EXPANDED, SSCI, A&HCI, ESCI Timespan=All years* |
| \| # 2 \| \| --- \| \| | [623,581](http://apps.webofknowledge.com.vu-nl.idm.oclc.org/summary.do?product=WOS&doc=1&qid=7&SID=F3hBnvbSnv7HrsVeRHI&search_mode=AdvancedSearch&update_back2search_link_param=yes) | TS=metasta* |
|  |  | *Indexes=SCI-EXPANDED, SSCI, A&HCI, ESCI Timespan=All years* |
| # 1 | [302,03](http://apps.webofknowledge.com.vu-nl.idm.oclc.org/summary.do?product=WOS&doc=1&qid=6&SID=F3hBnvbSnv7HrsVeRHI&search_mode=AdvancedSearch&update_back2search_link_param=yes) | TS=((colorectal OR colonic OR colon OR rectum OR rectal) NEAR/3 (cancer* OR carcinoma* OR tumor* OR tumour* OR neoplas*) ) |
|  |  | *Indexes=SCI-EXPANDED, SSCI, A&HCI, ESCI Timespan=All years* |

**Table 4: Wiley/Cochrane Library 57 results (9 November, 2020)**

| **ID** | **Search** | **Hits** |
| --- | --- | --- |
| #1 | (metastatic OR metastas*):ti,ab,kw | 41881 |
| #2 | ((colorectal OR colonic OR colon OR rectum OR rectal) NEAR/3 (cancer* OR carcinoma* OR tumor* OR tumour* OR neoplas*)):ti,ab,kw | 21364 |
| #3 | (locally NEXT advanced NEXT colorectal NEXT cancer* OR locally NEXT advanced NEXT colon NEXT cancer* OR locally NEXT advanced NEXT rectum NEXT cancer* OR locally NEXT advanced NEXT colorectal NEXT carcinoma* OR locally NEXT advanced NEXT colon NEXT carcinoma* OR locally NEXT advanced NEXT rectum NEXT carcinoma* OR locally NEXT advanced NEXT rectal NEXT cancer* OR locally NEXT advanced NEXT rectal NEXT carcinoma*):ti,ab,kw | 657 |
| #4 | (#1 AND #2) NOT #3 | 6353 |
| #5 | (HIPEC* OR IPC OR Hyperthermic NEXT intraperitoneal NEXT Chemotherap* OR Hyperthermic NEXT intra-peritoneal NEXT chemotherapy* OR peritoneal NEXT chemotherapy*):ti,ab,kw | 945 |
| #6 | #4 AND #5 | 57 |
